# Framework to function: mechanosensitive regulators of gene transcription

**DOI:** 10.1186/s11658-016-0028-7

**Published:** 2016-12-07

**Authors:** Megan Finch-Edmondson, Marius Sudol

**Affiliations:** 1 Mechanobiology Institute, National University of Singapore, 5A Engineering Drive 1, 117411 Singapore, Singapore; 2 Department of Physiology, National University of Singapore, Yong Loo Lin School of Medicine, 2 Medical Drive, 117597 Singapore, Singapore

**Keywords:** Mechanotransduction, Actin, Myocardin, MRTF, YAP, TAZ, β-catenin, Epithelial-mesenchymal transition

## Abstract

Mechanobiology has shifted our understanding of fundamental cellular and physiological functions. Changes to the stiffness of the extracellular matrix, cell rigidity, or shape of the cell environment were considered in the past to be a consequence of aging or pathological processes. We now understand that these factors can actually be causative biological mediators of cell growth to control organ size. Mechanical cues are known to trigger a relatively fast translocation of specific transcriptional co-factors such as MRTFs, YAP and TAZ from the cytoplasm to the cell nucleus to initiate discrete transcriptional programs. The focus of this review is the molecular mechanisms by which biophysical stimuli that induce changes in cytoplasmic actin dynamics are communicated within cells to elicit gene-specific transcription via nuclear localisation or activation of specialized transcription factors, namely MRTFs and the Hippo pathway effectors YAP and TAZ. We propose here that MRTFs, YAP and TAZ closely collaborate as mechano-effectors.

## Background

Mechanical signaling refers to the process by which a physical force such as pushing, pulling or shear stress can trigger a signaling event, which stimulates the transfer of information throughout the cell to elicit a response. The molecular mechanisms’ by which cells sense and respond to mechanical stimuli are referred to as mechanotransduction. Stretch-activated ion channels, integrin based cell-extracellular matrix (ECM) adhesions, cadherin based cell-cell contacts, receptors, cytoskeletal filaments as well as many other sensors and effectors have been shown to contribute to mechanotransduction. The cellular response to mechanical signals involves reorganization of the cytoskeleton, effecting cellular shape, orientation, polarity, migration, and gene expression.

Extracellular stimuli that alter actin dynamics are highly diverse and include soluble factors such as hormones and chemokines, or physical interactions between neighboring cells and the ECM. These signals are perceived by various receptor proteins including G protein-coupled receptors (GPCRs), Receptor Tyrosine Kinases (RTKs), and receptors for integrin, transforming growth factor-β (TGFβ), and E-cadherin signaling. Receptors link to Rho GTPases via selective Rho guanine nucleotide exchange factors (GEFs) that activate Rho proteins by catalyzing the exchange of GDP for GTP. Once activated, Rho GTPases regulate numerous downstream effector proteins to modulate actin polymerization chiefly via two well-established pathways, the first involving Rho-associated kinase (ROCK)–LIM kinase–cofilin signaling, and the other mediated by formins. Mammalian cells express at least 20 different Rho GTPases from eight subfamilies, the best-characterised being RhoA, Rac and Cdc42 (for a review of Rho GTPase signal transduction see [1, 2]).

Due to the complex nature of actin dynamics, adequate cellular response to extracellular stimuli not only requires polymerization and/or disassembly of actin filaments, but also coordinated synthesis of the myriad of structural proteins and regulatory factors that accompany this process. Cells must therefore be able to sense the status of actin cytoskeleton organization and be able to communicate this to the cell nucleus to regulate gene transcription. How this occurs in the cell remained a mystery until the seminal discovery that actin polymerization is the trigger for nuclear localisation of myocardin-related transcription factor (MRTF) to stimulate serum response factor (SRF)-dependent transcription [3]. Since then, other factors that respond to and actively regulate actin dynamics have been identified.

Whilst the function of cytoplasmic actin in regulating gene expression has been known for more than a decade, more recent investigations have shown that nuclear actin can also regulate gene transcription via its requirement for the activity of all three RNA polymerases, and its association with ribonucleoproteins and chromatin remodeling complexes (reviewed in [4]). Nuclear actin and its functional implication for general transcriptional activity will not be discussed here in detail. Rather this review will focus on how changes in cytoplasmic actin dynamics affect gene-specific transcription via nuclear localisation or activation of specialized transcription factors, namely MRTFs and the Hippo pathway effectors Yes-associated protein (YAP) and its paralog transcriptional coactivator with PDZ-binding motif (TAZ), in addition to some less characterised factors such as β-catenin, the NF-κB, Nrf2 and Foxj1a transcription factors, and epigenetic regulator HDAC3. Important to note is that in addition to their role in mechanotransduction, the transcription factors discussed in this review are involved in regulating various other cellular processes in response to alternate stimuli e.g., chemical ligand binding, and do not function solely as mechanotransducers.

## Myocardin-related transcription factor (MRTF) family

SRF is a member of the MADS-box family of transcription factors that was first described by Treisman in 1986. It is the factor that binds to the serum response element (SRE, or CArG sequence: CC[A/T]_6_GG) in the promoter region of c-fos to mediate cellular response following serum stimulation [5]. SRF is abundantly expressed in many cell types and directs the transcription of target genes in response to various signaling cascades. SRF target genes include ‘immediate-early’ genes, encoding for proteins required for re-entry into the cell cycle e.g., *c-fos* and *egr-1*, muscle specific genes e.g., alpha-actin and tropomyosin, regulators of actin dynamics and cell motility e.g., gelsolin and vinculin, and microRNAs (miR-1, miR-133a) (see review by [2]). Thus SRF is an important regulator of cellular function including growth, proliferation, migration, cytoskeletal organization and differentiation.

**Fig. 1:**
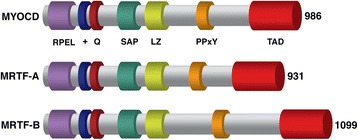
Schematic representation of the protein structure of the human myocardin-related transcription factor family. Various domains and motifs within the family members myocardin (MYOCD), myocardin-related transcriptional factor A (MRTF-A) and myocardin-related transcriptional factor B (MRTF-B) mediate specific functions: RPEL domain (*purple*) mediates cytoplasmic localisation and actin binding, basic (+; *blue*) and glutamine-rich (Q; *burgundy*) regions facilitate interaction with Serum Response Factor, whereas SAP domain (*green*) dictates promoter specificity. Leucine zipper (LZ; *lime*) mediates dimerization, and TAD (*red*) is a transcriptional activation domain. The PPxY motif (*orange*) mediates interaction with WW domains of partner proteins such as YAP. In MYOCD, PPSY is located at amino acid positions 768–771; in MRTF-A, PPGY is at amino acid positions 725–728; and in MRTF-B, PPRY is at amino acid positions 882–885. The number of amino acids for each protein is indicated

Myocardin (MYOCD), MRTF-A (MAL1/MKL1) and MRTF-B (MKL2/MAL16) are members of the MRTF family (Fig. 1) that interact with SRF to activate a panel of genes [6–8]. Notably, MRTFs exhibit different patterns of expression. Whilst myocardin is specifically expressed in cardiac and a subset of smooth muscle cells, MRTF-A and -B are expressed in a range of embryonic and adult tissues [8]. MRTFs also perform separate functions in vivo, revealed by knockout studies in mice. MYOCD-null mice survive only to embryonic day 10.5 (E10.5) exhibiting gross vascular defects likely due to blocked smooth muscle cell differentiation [9]. MRTF-B-null mice die slightly later at mid-gestation E14.5, with defects in cardiac outflow tract morphogenesis mimicking congenital heart disease [10, 11]. In contrast, MRTF-A is dispensable for normal development since MRTF-A-knockout mice are viable and fertile. This is surprising, since it is the most ubiquitously expressed of the MRTF family members. MRTF-A is however required for prolonged lactation, attributed to its role in differentiation and survival of myoepithelial cells, which are required for maintenance of lactation [12, 13].

Interestingly, despite sharing similar protein structure MRTF family members are subject to differential intracellular regulation. Whereas myocardin is usually localised to the cell nucleus, MRTF-A and -B are predominantly localised to the cytoplasm and only translocate to the nucleus following stimulation (reviewed in [14]). Nuclear translocation of MRTF after serum stimulation is controlled by Rho GTPases via actin dynamics (Fig. 3a). In a series of elegant experiments, Miralles et al. [3] showed that MRTF-A binds monomeric actin via three N-terminal RPEL motifs, effectively sequestering it in the cytoplasm. Rho-mediated actin polymerization releases MRTF, resulting in increased nuclear accumulation where it associates with SRF to drive transcription.

Treatment with drugs to alter the actin polymerization status provided evidence to support actin dynamics as the trigger for MRTF-A translocation and SRF activation. Latrunculin B, which impairs F-actin formation by sequestration of actin monomers, prevents nuclear accumulation of MRTF-A. The opposite effect was observed following treatment with cytochalasin D to promote actin dimerization [3]. These findings were recapitulated using overexpression of actin mutants that either favor (Val159Asn and Ser14Cys) or inhibit (Glu13Arg and Arg62Asp) actin polymerization [15]. Nuclear translocation of MRTF is also regulated in a serum-independent manner by the muscle-specific actin binding protein STARS (striated muscle activator of Rho signaling). STARS enhances actin polymerization, through a mechanism that requires its C-terminal actin-binding domain and RhoA, resulting in increased nuclear accumulation of MRTF [16]. Myocardin contains divergent RPEL1 and 2 motifs that have a lower affinity for actin compared to MRTF [17]. In contrast, binding of myocardin to the nuclear import machinery (the importin α/β1 heterodimer) (Fig. 3a) is stronger than that of MRTF-A/B [18]. Furthermore, access to two N-terminal leucine rich sequences that are required for CRM1-mediated nuclear export vary between myocardin and the MRTFs [19]. Taken together, these factors explain the differences observed between myocardin and MRTF-A/B subcellular localisation.

### MRTFs are mechanical sensors linking actin dynamics to SRF-mediated gene transcription

Before MRTFs were known to bind SRF to activate gene transcription, Sotiropoulos et al. [20] showed that SRF activation by the actin regulator LIM kinase-1 (LIMK1) is dependent on its ability to promote F-actin stabilization via phosphorylation of cofilin. Activation of SRF by actin dynamics is sufficient to induce transcription of *vinculin*, cytoskeletal *actin* and *srf* itself. Using *Srf*-null embryonic stem cells, Schratt et al. [21] demonstrated that cell spreading, adhesion and migration is impaired by loss of SRF, due to an inability to form focal adhesion plaques and stress fibers. Consistent with previous reports identifying MRTF-A as the mediator of SRF activation in response to actin dynamics in mammals [3], analysis of border cell migration during *Drosophila* oogenesis revealed nuclear localisation of MAL-D (*Drosophila* ortholog of MRTF) correlates with the stretched shape of migrating cells [22]. Moreover, nuclear localisation of the MAL-D/SRF complex is required to establish a robust F-actin cytoskeleton, necessary for invasive migration [22]. The authors’ propose that tension-induced MAL-D nuclear accumulation may provide positive feedback regulation for cytoskeletal actin dynamics and migration.

Using collagen coated magnetic beads the McCulloch group applied static tensile forces to cultured cardiac fibroblasts to further study MRTF regulation by mechanical stress. The applied force induced Rho-dependent actin assembly, promoting nuclear translocation of MRTF and activation of SRF-dependent gene transcription as determined by α-smooth muscle actin (α-SMA) expression [23]. In a comprehensive report, nuclear accumulation of MRTF-A stimulated by serum, actin drugs or mechanical stress was blocked in cells maintained at tensional homeostasis [24]. Tensional homeostasis refers to the situation in which there is a balance between the external (ECM) and internal (cytoskeletal) forces. This was achieved by plating cells on mechanically loaded, anchored matrices, and was accompanied by a higher G/F-actin ratio, mediated by increased cofilin expression. From these studies it is clear that because MRTFs can respond directly to changes in actin dynamics, any situation that exposes cells to mechanical forces will elicit a robust transcriptional response mediated by MRTF/SRF signaling.

### MRTFs are “master regulators” of epithelial-mesenchymal transition (EMT)

Epithelial–mesenchymal transition (EMT) is a cellular phenotypic shift accompanied by changes in gene expression of numerous transcription factors and cytoskeletal proteins that enable cells to dissociate their cell–cell contacts and migrate. EMT governs a variety of developmental processes including gastrulation, neural crest development, and heart valve formation (reviewed in [25]). EMT also plays a significant role in the development of pathological conditions, namely organ fibrosis and cancer progression. Increased ECM rigidity is a hallmark of fibrosis and metastasis, and mechanical tension has been identified as a regulator of EMT. Due to their role in regulating and responding to changes in the actin cytoskeleton, it is not surprising that the MRTFs are implicated in EMT.

TGFβ is a major inducer of EMT, acting via several different mechanisms including SMAD-dependent and -independent signaling pathways [26]. TGFβ triggers the Rho-dependant nuclear localisation of MRTF, which forms a complex with Smad3 to induce transcription of *slug*, a repressor of E-cadherin and positive regulator of EMT [27]. Moreover, MRTFs increase expression of actin cytoskeletal proteins (caldesmon, tropomyosin and β-actin) to induce reorganization of the cytoskeleton, effectively operating as a feed-forward mechanism for MRTF-activation. Disruption of cell-cell junctions by removal of calcium is also sufficient to enhance nuclear accumulation of MRTF-A and SRF, leading to activation of α-SMA, a marker of cells that have transdifferentiated to the myofibroblast phenotype [28]. A 2010 study by Gomez et al. found that a sheet of mammary epithelial cells treated with TGFβ displayed variability in expression of EMT markers [29]. Investigation of the relative cellular forces across the cell sheet revealed that cells within regions experiencing the highest mechanical stress preferentially underwent EMT. Because nuclear localisation of MRTF-A correlates directly with mechanical stress, tissue geometry and the resultant variability in cytoskeleton dynamics dictates EMT responsiveness following TGFβ stimulation via regulation of MRTF activation. Along the same lines, restriction of cell spreading [30] and decreased matrix rigidity [31] both prevent MRTF-A nuclear translocation and block transdifferentiation. These studies provide a clear link between mechanical stress, MRTF-A translocation and EMT, and contribute to our understanding of the complex nature of how biophysical cues influence biological outcome.

### Role of MRTFs in fibrosis and cancer

Aberrant EMT activation underlies development of tissue fibrosis and cancer progression [25]. Due to its role in regulating EMT, MRTF-A has been linked to multiple pathologies including lung and liver fibrosis, and metastasis in a variety of human cancers. Increased nuclear MRTF-A was observed in a mouse model of lung fibrosis (intratracheal bleomycin) and samples from patients with idiopathic pulmonary fibrosis [32]. Functionally, inhibition of MRTF-A mechanosignaling via treatment with the ROCK inhibitor fasudil during the fibrotic stage of lung injury, or genetic ablation of MRTF-A, protected mice from experimental lung fibrosis [32]. Similarly, knockout of MRTF-A significantly reduced carbon tetrachloride (CCl_4_)-induced liver fibrosis in mice [33]. MRTF-A null mice exhibited a suppressed hepatic stellate cell response as determined by reduced hepatic stellate cell activation markers e.g., type I collagen (Col1a) and α-SMA [33]. This finding is significant since in the majority of cases, chronic liver injury characterised by liver fibrosis precedes the development of primary liver cancer.

Increased *MRTF-A* RNA expression correlates with breast cancer metastasis in human patient samples [34]. MRTF-A, together with STAT3, promotes migration of MDA-MB-231 breast cancer cells via up-regulation of Myl9 and Cyr61 [34]. Myl9, a component of the actomyosin contractile apparatus, and the ECM-associated signaling protein Cyr61 have both been implicated in the invasive characteristics of tumour cells [35, 36]. As in breast cancer, MRTF-A expression correlates with a more invasive lung cancer phenotype [37]. Depletion of MRTF decreased in vitro and in vivo migration and invasion, likely due to repression of matrix metalloproteinase 9 (MMP9) expression [37], an MRTF-A target that has been implicated in lung tumorigenesis [38].

In the pancreas, increased MRTF-A and –B expression promotes generation of stem cell-like cells from normal cells via up-regulation of microRNAs associated with EMT and cancer initiating cells [39]. Overexpression of MRTF-A and –B promoted pancreatic cancer growth in a nude mouse assay, and high expression of MRTFs in pancreatic cancer cell lines is associated with resistance to the chemotherapeutic agent gemcitabine [39]. Alteration towards a more stem cell-like phenotype and increased drug resistance is meaningful since less differentiated tumours tend to be more aggressive and typically respond poorly to traditional chemotherapeutics [40].

### Therapeutic targeting of MRTF-A

Accumulating evidence highlighting MRTF-A as a mediator of fibrotic disease and metastasis suggests that targeting MRTF-SRF signaling for therapy could be beneficial. CCG-1423, a small molecule inhibitor of RhoA signaling [41], inhibits nuclear accumulation of MRTF-A by blocking its interaction with importin α/β1 through binding to the N-terminal basic domain of MRTF-A [42]. This discovery paved the way for development of second-generation compounds that have improved cytotoxicity e.g., CCG-100602 and CCG-203971 [43]. Using two in vitro models of intestinal fibrogenesis treatment with second-generation MRTF-A inhibitors was able to block both physical (matrix stiffness-induced) and biochemical (TGFβ-induced) fibrogenesis [43]. MRTF-A inhibition reduced expression of actin contractile (*Mylk*) and fibrogenic (*Col1a*) genes and α-SMA protein expression.

Important to note however, is that myofibroblast differentiation is a normal physiological response to injury. During wound healing keratinocytes gain mesenchymal features to enable migration and re-epithelialisation [44]. Similarly, cardiac remodelling following myocardial infarction requires differentiation of fibroblasts to myofibroblasts, and this process is regulated by MRTF-A [45]. Increased MRTF-A activation could therefore be harnessed therapeutically to accelerate the wound healing process. The small molecule isoxazole (ISX) was previously shown to promote myofibroblast differentiation of cardiac progenitor cells [46]. Subsequently, ISX was found to stimulate MRTF-A dependent gene expression via regulation of MRTF-A stability, though the mechanism for this is unclear [47]. Importantly, treatment of dermal biopsies in mice with ISX significantly accelerated wound closure and suppressed the inflammatory response [47], indicating that modulation of MRTF-A activity is a feasible option to promote wound healing in humans.

### SRF-independent roles of MRTF-mechanosignaling

The function of MRTF as a mechanosensor is not completely reliant on its interaction with SRF. Tenascin-C (TNC) is an ECM protein that is highly expressed in tissues experiencing increased mechanical stress such as tissue remodeling, wound healing and tumorigenesis (reviewed in [48]). Investigation of the mechanism of TNC up-regulation in response to mechanical stress identified a SAP domain-dependent, SRF-independent interaction of MRTF-A with the TNC promoter [49]. In a follow-up publication the same group identified a set of breast cancer specific genes, including TNC, that are regulated by MRTF-A in an SRF-independent manner [50]. Expression of this gene set is implicated in regulation of cellular proliferation, motility and cancer, and correlates with poor patient prognosis [50].

More recently, MRTF-A has been implicated in the regulation of promoter methylation status to control gene transcription. MRTF-A coordinates Histone H3 Lysine 4 (H3K4) methylation on the MMP9 promoter to drive lung cancer cell migration and invasion [37]. H3K4 methylation is catalyzed by the COMPASS/COMPASS-like methyltransferase complex, and MRTF-A recruits ASH2, a member of this complex, to activate MMP9 transcription [37] (Fig. 3a). Similarly in activated stellate cells, MRTF-A recruited ASH2 to fibrogenic gene promoters (e.g., Col1a1, Col1a2 and Acta2) to activate their transcription and switch on a pro-fibrogenic transcriptional program [33]. Silencing of COMPASS components significantly down-regulated the expression of MRTF-A target genes and blocked experimental liver fibrosis in mice [33]. The discovery that MRTF can regulate gene expression epigenetically will no doubt lead to the identification of novel MRTF-regulated target genes, and adds to our understanding of the complex mechanisms governing mechanotransduction.

## The Hippo signaling pathway

**Fig. 2:**
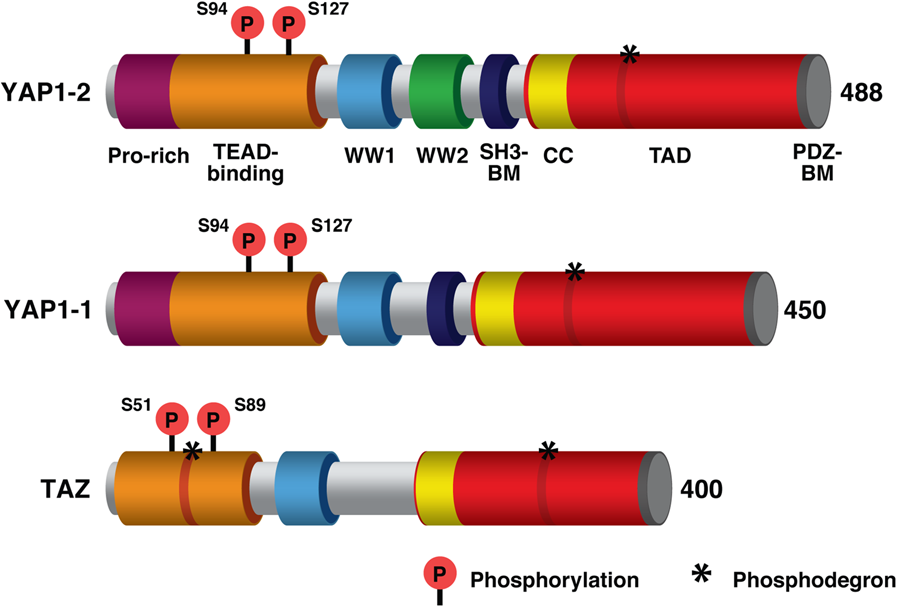
Schematic representation of the protein structure of the single (YAP1-1) and double (YAP1-2) WW domain isoforms of human YAP, and TAZ. YAP harbors a proline-rich region (Pro-rich; maroon) at its N-terminus which is lacking in TAZ. DNA-binding is primarily mediated by interaction with TEAD proteins via the TEAD-binding domain (*orange*), with phosphorylation on serine residue 94/51 in YAP and TAZ respectively important for this interaction. WW domains (WW1; *light blue* and WW2; *green*) mediate protein-protein interactions with PPxY containing partners including LATS and MRTFs [149] whereas the SRC homology 3 binding motif (SH3-BM; *dark blue*) enables YAP’s association with the SH3 domain of Yes and Src protein-tyrosine kinases. The transcriptional co-activator activity of YAP/TAZ is mediated by a strong transcriptional activation domain (TAD; *red*) that contains a coiled-coil (CC; *yellow*) motif. Nuclear localisation of YAP/TAZ is mediated by a Post-synaptic density, Discs large, Zonula occludens-1-binding motif (PDZ-BM; *dark grey*) [150]. Phosphorylation of serine 127/89 on YAP and TAZ respectively promotes their cytoplasmic sequestration facilitated by interaction with 14-3-3-proteins. YAP and TAZ also contain phosphodegron sequences (*) whereby phosphorylation of specific residues marks YAP and TAZ for degradation by the proteasome. The number of amino acids for each protein is indicated

The Hippo signaling pathway is a complex network of proteins that control organ size via regulation of cellular proliferation, survival and differentiation. Initially discovered by genetic mosaic screens in *Drosophila*, the core of the Hippo pathway comprises a pair of highly conserved kinases and their adaptor proteins that, in mammals, centers on two effectors: YAP [51] and TAZ (also known as WWTR1) [52] (Fig. 2). YAP and TAZ are potent transcriptional coactivators that associate with various DNA-binding proteins e.g., TEAD factors, to drive gene transcription. For a comprehensive review of the Hippo pathway, its regulators and physiological functions, the reader is directed to two excellent reviews [53, 54].

**Fig. 3:**
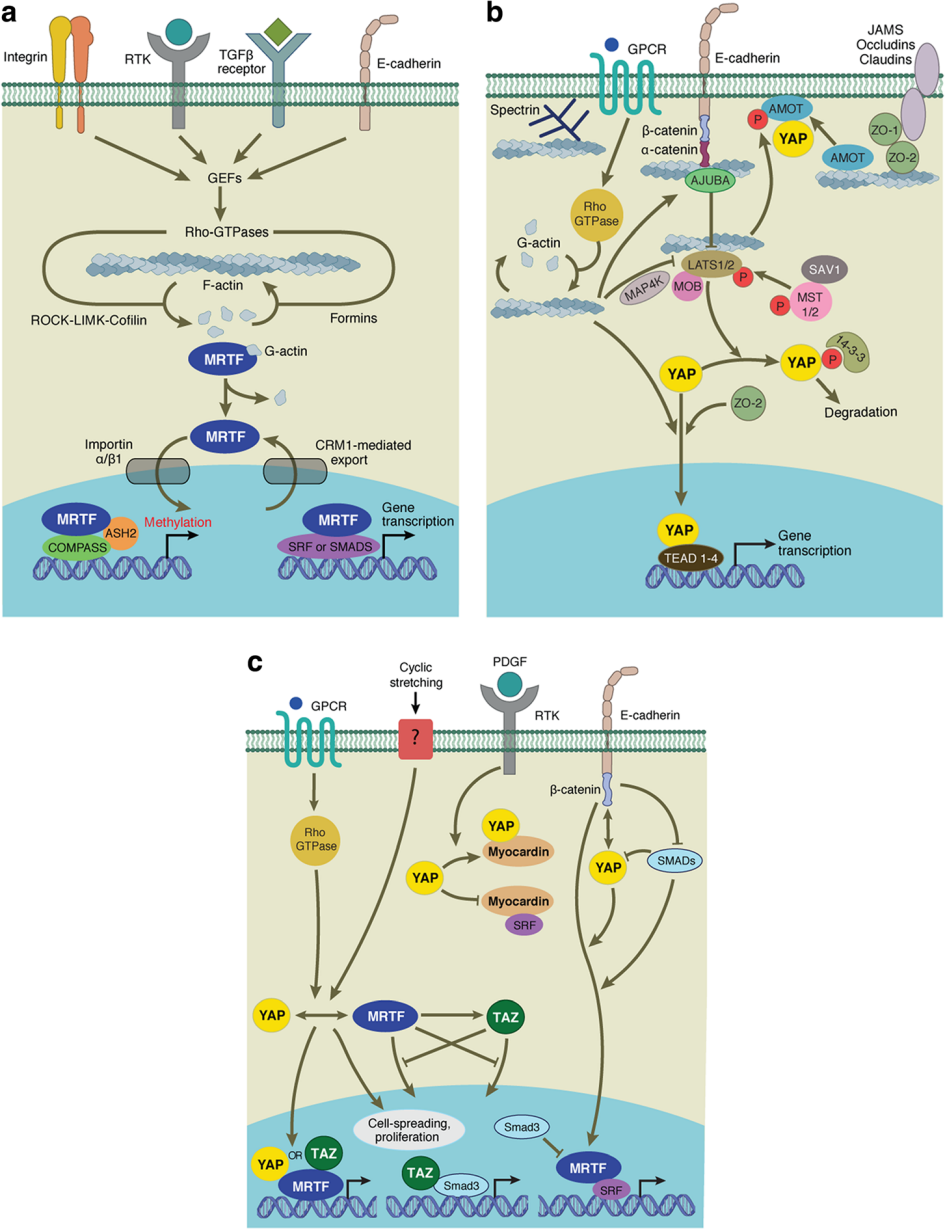
Mechanosensitive regulators of gene transcription. Signaling diagrams showing mechanisms linking mechanical cues to **a** myocardin-related transcription factor (MRTF) family mediated gene expression, **b** Hippo-YAP/TAZ activity, and **c** crosstalk between the mechanosensing mediators discussed in this review

Triggered by various upstream stimuli, for example cell-cell contact [55], the MST1/2 kinases together with the adaptor protein SAV1 (WW45) phosphorylate and activate LATS1/2 and MOB [56, 57]. Activated LATS then phosphorylates YAP and TAZ on specific serine residues [58–60]. Phosphorylation of Ser127 and Ser89 of YAP and TAZ, respectively, generates a 14-3-3-protein binding site resulting in their cytoplasmic sequestration [52, 61]. In addition, LATS phosphorylation on alternate residues marks YAP and TAZ for degradation by the proteasome [62, 63] (Fig. 3b). Activation of the Hippo signaling pathway thus inhibits YAP and TAZ activity. Mechanisms coupling extracellular signals with the core Hippo kinase cassette are complex and not yet completely understood. Recently, mechanical cues from the cytoskeleton including cell density, substrate stiffness, cellular tension, and GPCR signaling have been identified as regulators of YAP/TAZ activity (Fig. 3b) (reviewed by [64, 65]).

### The Hippo pathway effectors YAP and TAZ respond robustly to mechanical cues

Early indications that YAP/TAZ activity is regulated by mechanical cues came from the important observation that YAP localisation and phosphorylation status is regulated by cell density [55]. In sparsely populated cells YAP is predominantly localised to the nucleus and in its active un-phosphorylated form. Contrastingly, in high density culture YAP is phosphorylated and localised to the cytoplasm, and this process is regulated by Hippo pathway signaling [55]. A change in cell density alters both cell-cell contact (adhesion) and cell morphology. To investigate regulation of the Hippo pathway by cell morphology, independent from cell adhesion, Wada et al. [66] grew single cells on variously sized fabricated micropatterned cell adhesive areas (called microdomains). In cells grown on small domains YAP is mostly cytoplasmic, whereas YAP localised to the nucleus on large domains [66]. Cell morphology-induced YAP localisation is dependent on LATS activity, indicating that cell morphology is a Hippo pathway regulator.

YAP/TAZ localisation and activity is also controlled by the rigidity of the ECM. On hard substrates YAP and TAZ are predominantly nuclear and become increasingly cytoplasmic on softer substrates [67]. Importantly, ECM rigidity not only influences YAP/TAZ activity in isolated cells, but also in confluent monolayers. Similarly, analysis of YAP/TAZ localisation within a three-dimensional cell sheet demonstrated that variations in mechanical stress pattern YAP/TAZ nuclear localisation, where high stress correlates with increased nuclear localisation, and vice versa [68]. The latter study also revealed that mechanical stretching of contact inhibited cells i.e. exhibiting cytoplasmic YAP, is sufficient to induce YAP/TAZ re-entry into the nucleus to stimulate cellular proliferation [68]. This is important since it shows that mechanical stress is capable of overcoming inactivation of YAP/TAZ by Hippo pathway signaling. Interestingly, all of these studies identified actin cytoskeletal reorganization as a dominant regulator of YAP and TAZ.

In support of this, a functional connection between GPCR/Rho signaling, cytoskeletal reorganization, and YAP/TAZ activity has been elucidated. In response to chemical stimuli (e.g., LPA; lysophosphatidic acid and S1P; sphingosine 1-phosphophate) YAP and TAZ are dephosphorylated and enter the nucleus [69, 70]. Notably, the status of F-actin polymerization correlates with YAP activation. Similarly, YAP activity is regulated by cell attachment/detachment and this is mediated by Rho deactivation and cytoskeletal reorganization [71]. Indeed, YAP/TAZ inactivation is responsible for cell detachment-induced anoikis, which is a specific type of apoptosis [71]. In these studies the LATS kinases were found to be the major regulator of YAP/TAZ activity in response to GPCR stimulation [69, 71], though intriguingly, GPCR signaling can either activate or inhibit YAP activity depending on the particular G protein coupled to the receptor [69]. In general we can consider that increased Rho GTPase activity and actin polymerization activates, whereas destabilisation of actin inhibits, YAP and TAZ (Fig. 3b).

### Mechanisms linking mechanical signals to YAP/TAZ activity

Unlike MRTF, YAP and TAZ are not known to directly bind actin; rather YAP and TAZ response to mechanical cues is controlled by actin binding proteins that are recruited to, and regulated by, the cytoskeleton. As alluded to above, actin polymerization and particularly the formation of stress fibers is a chief regulator of YAP/TAZ activity. In *Drosophila* imaginal discs, induction of F-actin formation by depletion of capping proteins A or B, or *capulet* (which sequesters actin monomers) induced a strong overgrowth phenotype via increased nuclear localisation of Yorkie (Yki, *Drosophila* YAP ortholog) [72, 73]. Inhibition of stress fiber formation by treatment with latrunculin A and cytochalasin D prevents nuclear accumulation of YAP/TAZ and abolishes their transcriptional activity following a range of stimuli such as cell attachment and manipulation of cell morphology [66, 67, 69, 71]. Moreover, depletion of F-actin-capping and -severing proteins (CapZ, Cofilin, and Gelsolin) is sufficient to induce YAP/TAZ nuclear localisation and gene expression in high density cell cultures in which YAP/TAZ have been inactivated [68]. Remarkably, while some studies found mechanical regulation of YAP/TAZ to be independent of the Hippo pathway [67, 68, 70], others show that the LATS kinases are essential [66, 69, 71].

Whether there truly are both Hippo-dependent and -independent mechanisms linking stress fibers to YAP activity is unclear. Indeed it remains to be elucidated even how LATS activity is regulated by actin polymerization. Recently, the Ste-20 kinase Happyhour and its mammalian counterparts MAP4K1/2/3/5 were found to regulate LATS activity in response to F-actin polymerization via direct phosphorylation of its hydrophobic motif [74] (Fig. 3b). This corroborates previous data demonstrating LATS Ser909 and Thr1079 phosphorylation is altered by GPCR signaling [69], and justifies the dispensable nature of MST1/2 for YAP/TAZ mechano-regulation, though the link between actin polymerization and MAP4K1/2/3/5 activation remains to be determined.

A mechanism linking mechanical forces to LATS was identified in *Drosophila* wing imaginal discs. In response to increasing cytoskeletal tension Jub, the ortholog of mammalian Ajuba and a negative regulator of Warts (*Drosophila* LATS ortholog), preferentially localises to apical junctions via its association with α-catenin, an actin associated protein [75]. Jub is a negative regulator of Warts and recruits Warts to junctions in a tension-dependent manner (Fig. 3b). The outcome of this is that increased tension within the cytoskeleton increases *Drosophila* wing growth due to increased Yki activity and vice versa [75]. A second study from the same group identified inhibition of LATS by LIMD1, another member of the mammalian Ajuba protein family, as the mechanism linking cyclic stretching and YAP activity in mammalian cells [76]. Mechanical strain activates c-Jun N-terminal kinase (JNK) [77]. Using specific JNK inhibitors and shRNA-mediated depletion the authors observed that JNK activates YAP activity in response to cyclic stretching [76]. Phosphorylation of LIMD1 by JNK increases it’s binding to LATS, effectively blocking YAP/TAZ phosphorylation.

The role of LATS in transducing mechanical signals to YAP/TAZ is complicated by the fact that Hippo signaling exhibits feedback to influence actin assembly. F-actin accumulates abnormally in *Drosophila* when Hippo pathway activity is reduced or abolished, independently of Yki activity [73]. Zyxin, a promoter of actin polymerization that is regulated by mechanical forces [78], has been shown to interact directly with Warts/LATS in at least two studies. FAT, a cadherin transmembrane receptor, regulates localisation of the myosin Dachs, which subsequently binds zyxin and stimulates its binding and inhibition of Warts [79]. Separate to its role in the Hippo pathway, LATS targets phosphorylated zyxin to the mitotic apparatus to regulate actin dynamics during mitosis [80]. Interestingly, zyxin can also promote the interaction of Yki and Scalloped (*Drosophila* TEAD ortholog) to drive Yki target gene expression and tissue growth [81]. Moreover, yet another study found that LATS is a novel actin binding protein that can directly inhibit actin polymerization [82]. Hence involvement of the Hippo pathway in actin-mediated YAP/TAZ regulation is multifaceted, and it is likely that LATS participates via more than one mechanism simultaneously.

Important to highlight is the recent report by Das et al. [83] that purports the uncoupling of phosphorylation and F-actin mediated nuclear localisation of YAP in non-contact inhibited cells. Specifically, in sparsely populated cells, the authors observed that despite increased phosphorylation of YAP upon inhibition of actomyosin contractility (by treatment with blebbistatin), YAP protein remained localised to the nucleus, including phosphorylated YAP [83]. This was in stark contrast to cells treated with latrunculin A (to de-polymerize actin), in which YAP was effectively excluded from the nucleus, even when a LATS phosphorylation-insensitive mutant (Ser127Ala equivalent) was utilised. These novel results suggest that the control of YAP localisation by actin polymerization/de-polymerization can override the canonical Hippo pathway-mediated regulation of YAP.

Angiomotins (AMOTs) are known regulators of YAP/TAZ localisation and activity via Hippo-dependent [84] and –independent [85] mechanisms. AMOTs bind to F-actin, and in response to perturbations of the actin cytoskeleton, dissociate from actin to bind and sequester YAP in the cytoplasm [86] (Fig. 3b). Activated Hippo pathway signaling further enhances this process, since phosphorylation of AMOT by LATS inhibits its F-actin binding to promote YAP cytoplasmic localisation [87]. Interestingly, AMOTs are required for regulation of YAP localisation induced by a number of stimuli including increased cell density, treatment with actin depolymerizing drugs, or GPCR activation by serum withdrawal [86]. Hence AMOTs are yet another group of proteins that connect F-actin architecture to YAP/TAZ regulation.

The spectrin network is one of the most recently identified regulators of YAP/TAZ activity in response to mechanical stimuli. Spectrin functions as scaffold protein at the membrane–cytoskeleton interface via cross-linking of short F-actin filaments, and can bind integral membrane proteins (reviewed in [88]) (Fig. 3b). Reports from three different groups identified spectrin as a regulator of Yki/YAP in *Drosophila* and mammalian cells [89–91]. Mutation or depletion of spectrin subunits in *Drosophila* induces Yki-dependent cell polarity defects or tissue overgrowth. Although one study found that dysregulation of apical spectrin alters the activity of the upstream Hippo pathway regulator Expanded [90], the consensus appears to be that the basolateral spectrin network regulates cortical actomyosin tension, potentially via phosphorylation of non-muscle myosin II [89], which in turn regulates Yki/YAP/TAZ activity by an as yet unidentified mechanism. Notably, spectrin does not alter Ajuba/Warts localisation to apical junctions [89–91], nor is there evidence for the involvement of JNK activation [90].

Integration of the wide array of biochemical and mechanical cues encountered by a cell is complex, and under ever-changing conditions can be extremely difficult to consolidate. In the report from Sun et al. [92], the authors present a computational model that integrates multiple components involved in mechanotransduction including adhesion complexes, intracellular signal transmission, and cytoskeleton dynamics, with known regulatory pathways directing transcriptional programs such as Hippo-YAP/TAZ and SRF/MRTF signaling [92]. Using this model, the effect of changes in various signaling molecules on YAP/TAZ activity can be predicted, revealing for example that overexpression of the adhesion molecule FAK is expected to increase YAP/TAZ activity in cells plated on soft (e.g., 20 kPa) substrates [92]. Notably, the model also predicts that YAP/TAZ is more sensitive to changes in ECM properties than SRF/MRTF [92]. This is an intriguing prediction that is in contrast to the observation that MRTF-A translocated to the nucleus three times faster than YAP in response to cyclic stretching of primary mouse embryonic fibroblasts [93]. Given the relatively recent arrival of YAP/TAZ in the field of mechanotransduction, there is no doubt researchers will endeavor to fully delineate the differences and similarities between MRTF and YAP/TAZ experimentally.

### Biological outcomes of YAP/TAZ mechanotransduction: development and differentiation

As introduced above, the Hippo pathway is a critical regulator of organ size during development and tissue homeostasis in the adult. Furthermore, dysregulation of Hippo signaling underlies the development and progression of numerous types of human cancer. It is not surprising therefore that mechanical signaling has been linked to the regulation of YAP/TAZ activity in a variety of biological contexts in particular cellular differentiation, fibrosis and cancer cell invasion. Specification of the trophectoderm and inner cell mass lineages in the mouse blastocyst correlates with cell polarization and YAP localisation [94, 95]. Trophectoderm derives from outer cells where YAP is nuclear and actively promoting transcription of trophectoderm-specifying genes. Inhibition of Rho-ROCK signaling during the early stages of embryogenesis results in activation of the LATS kinases [96]. The subsequent reduction in nuclear localised YAP correlates with mislocalisation of key components of the apical-basal cell polarity, and impairs trophectoderm formation [96].

Truncation of YAP in the medaka fish *hirame* (*hir*) mutant results in a markedly flattened body characterised by tissue flattening and misalignment [97]. YAP knockdown in wild-type embryos recapitulated the *hir* phenotype, and the phenomenon could be imitated with human cells using an in vitro three-dimensional spheroid culture system. The Rho GTPase activating protein ARHGAP18 was identified as an effector of YAP that controls actomyosin-mediated tissue tension [97]. This study identifies YAP as essential for the attainment of proper three-dimensional body shape. Remarkably, the orientation of body flattening correlated with the direction of gravity. Thus perhaps YAP is the long sought after sensor of gravity proposed nearly a century ago by D’Arcy Thompson [98]. Either way, these studies show that from the very early stages of development, YAP, and most likely TAZ, is essential for proper development/differentiation.

Mechanical signal regulation of YAP/TAZ is also strongly linked to cell fate determination of multiple lineages, in particular neuronal and osteogenic differentiation. Studies from two groups found that culture of human pluripotent stem cells (hPSCs) on compliant versus rigid substrates markedly improved differentiation of hPSCs to post-mitotic motor neurons [99, 100]. Inhibition of nuclear localised YAP by LATS activation was identified as the driving factor for increased neuronal differentiation on soft surfaces. Disruption of actin dynamics or depletion of YAP is sufficient to stimulate neuronal differentiation on rigid surfaces [99] whereas LATS1 knockdown inhibited differentiation on soft surfaces [100]. YAP/TAZ associate with phosphorylated SMADs to inhibit their nuclear localisation and maintain cellular pluripotency [101]. Interestingly, Sun et al. [100] observed decreased phosphorylation and co-localisation of SMADs with YAP/TAZ on soft substrates. Thus the mechano-regulated interplay between YAP/TAZ and SMADs is likely to be important for rigidity-dependent neuronal differentiation.

Similarly, the fate of mesenchymal stem cells is regulated by substrate density [102], though remarkably modulation of YAP/TAZ abundance can switch the outcome of differentiation. For example, YAP/TAZ knockdown enabled adipogenic differentiation on stiff substrates that would usually produce osteoblasts [67]. The consequence of this can be observed in a practical example where microgravity (weightlessness) induces observed bone loss of approximately 1–2% per month in space. Osteogenic differentiation of bone marrow derived mesenchymal stem cells was inhibited in cells grown in a clinostat to simulate microgravity [103], and this correlates with dramatically decreased TAZ RNA and protein expression. Inhibition of osteogenesis could be overcome by stimulation of GPCRs with LPA to activate Rho-TAZ signaling, indicating this pathway may be therapeutically targeted to prevent bone loss during space flight.

### Biological outcomes of YAP/TAZ mechanotransduction: fibrosis and cancer

Like the MRTFs, YAP and TAZ have been implicated as key pro-fibrogenic regulators. Fibrotic lung [104] and liver [105] exhibit increased YAP/TAZ staining due to a marked increase in high YAP/TAZ expressing spindle-shaped fibroblasts. These cells exhibit pronounced nuclear localisation of TAZ [104] or YAP [105], which correlates with characteristic fibroblastic functions in vitro including proliferation, matrix synthesis, contraction and proliferation. Indeed YAP is essential for fibroblast activation: siRNA-mediated YAP/TAZ knockdown or treatment of cells with verteporfin, an inhibitor of YAP that disrupts the YAP/TEAD complex, blocked induction of cell spreading, actin polymerization and fibrogenic gene expression (e.g., *Acta2* and *Col1a1*) in response to activating culture conditions [104, 105]. Further, treatment of mice with verteporfin is able to ameliorate fibrosis in mice injected with CCl_4_ [105]. In lung fibrosis, plasminogen activator inhibitor-1 (encoded by *SERPINE1*) was identified as a YAP/TAZ target gene that promotes cell-matrix adhesion and continual YAP/TAZ activation [104]. Thus YAP and TAZ operate in a fibrotic positive-feedback loop, resulting in persistent cellular activation and pathological fibrosis.

Activation of YAP and TAZ has long been associated with tumorigenesis due to up-regulation of oncogenic gene targets promoting proliferation and resistance to apoptosis. Recent evidence suggests that cancer progression mediated by YAP/TAZ is due to its role in promoting matrix stiffness, cancer cell invasion and angiogenesis. Cancer associated fibroblasts are found in many solid tumours and promote the growth and invasion of cancer cells by various mechanisms (see review by [106]). Notably, activation of YAP (and most likely TAZ) is required for cancer associated fibroblast function [107]. YAP induces the expression of several cytoskeletal regulators such as *ANLN* and *DIAPH3* to promote ECM remodeling and invasion. Consistent with this, nuclear accumulation of YAP positively correlates with more advanced and aggressive human breast tumours with increased ECM rigidity indicated by linearization (cross-linking) of collagen bundles [108].

Resistance to chemotherapeutic agents is another trait of cancer cells exhibiting increased YAP/TAZ activation. Studies of breast cancer cells found that increased *TAZ* expression correlates with resistance to traditional chemotherapeutics paclitaxel and doxorubicin [109, 110]. Moreover, silencing of TAZ in xeno-transplanted human breast cancer stem cells significantly increased the efficiency of chemotherapy in vivo [111]. Similar observations were made when assessing the link between YAP abundance and cetuximab resistance in colorectal cancer patients [112]. Recently, using BRAF mutant melanoma cell lines, Kim et al. [113] showed an increase in nuclear accumulation of YAP/TAZ, accompanied by a concomitant increase in stress fiber formation, during the development of vemurafenib resistance. This result is important since it is the first to link actin dynamics and subsequent YAP/TAZ regulation to the acquisition of drug resistance. These findings indicate that down-regulation of TAZ/YAP expression or inhibition of actin remodeling in tumours, coupled with- or prior to- administration of chemotherapy, may have significant therapeutic value.

## Additional mediators of actin-regulated gene transcription

Whilst MRTFs and YAP/TAZ are the most well characterised actin regulated transcription factors, several additional mechanically regulated factors have been identified including β-catenin, the NF-κB, Nrf2 and Foxj1a transcription factors, and epigenetic regulator HDAC3. Cadherin-catenin complexes are responsible for mediating cell-cell adhesion (e.g., adherens junctions) and typically comprise classical cadherins such as E-cadherin, β-catenin, and α-catenin, which facilitates binding to vinculin, α-actinin and actin. Cadherin-catenin complexes participate in mechanosignaling by transmission of actomyosin-generated forces throughout a tissue (reviewed in [114]). β-catenin is a transcriptional co-activator whose activity is hypothesised to be regulated by recruitment and release from cadherin complexes. This is supported by the finding that overexpression of activated ROCK2 in mouse skin results in stiffness-mediated activation of β-catenin characterised by translocation from cell surface E-cadherin to the nucleus, and up-regulation of β-catenin target genes, in particular Cyclin D1, to drive epidermal hyperproliferation and consequent skin thickening [115]. Importantly, inhibition of actomyosin contractility or deletion of β-catenin could abolish the effects of ROCK-overexpression.

Mechanical stretching of lung parenchyma increases activation of NF-κB and AP-1 transcription factors via stretch-activated channels [116]. Activation of MAP kinase signaling, a known regulator of NF-κB and AP-1, were responsible for their increased activity. Moreover, NF-κB mediates up-regulation of cyclooxygenase-2 [116], a pro-inflammatory gene associated with asthma that is also increased by mechanical stretch of uterine myocytes in vitro [117] and during pregnancy and labour. Fluid shear stress stimulates increased protein expression and nuclear localisation of the Nrf2 transcription factor in endothelial cells in a phosphatidylinositol 3-kinase-dependent manner [118]. Shear stress induces expression of Nrf2 target gene *heme-oxygenase 1*, which is an antioxidant known to offer protection from development of atherosclerotic lesions in regions of high fluid shear stress (reviewed by [119]). Moreover, in response to epithelial distension and stretch caused by increased fluid pressure, the Foxj1a transcription factor is activated, mediating cilia motility in zebrafish [120].

In addition to gene specific activation in response to altered cellular tension, increased actomyosin contractility correlates with increased levels of global histone H3 lysine 9 acetylation, a marker of transcriptional activation [121]. Interestingly, perturbation of actomyosin contractility by treatment with blebbistatin, latrunculin A or cytochalasin D results in cytoplasmic-to-nuclear redistribution of HDAC3 and subsequent reduction in global histone acetylation levels [121]. This phenomenon is hypothesised to involve the acytomyosin-dependent stabilization of IκB-α, which binds and sequesters HDAC3 in the cytoplasm. Thus actin dynamics play a crucial role in the regulation of global gene expression via maintenance of an acetylated “active” chromatin structure.

## Crosstalk and cooperation of mechanotransduction pathways

Several publications have identified crosstalk and cooperation between the mechanosensing pathways covered by this review (Fig. 3c). YAP negatively regulates myocardin expression as well as its association with SRF to control the phenotypic switch of vascular smooth muscle cells in response to stimulation with platelet-derived growth factor. Overexpression of YAP inhibited contractile gene expression including *α-SMA*, *SM22α*, *SMMHC* and *MYOCD* itself, whilst promoting transcription of pro-proliferative genes [122]. YAP was found to specifically interact with myocardin, which reduced its co-immunoprecipitation with SRF, hence reducing SRF-directed transcription of smooth muscle genes (Fig. 3c). YAP therefore plays a functional role in controlling the vascular smooth muscle cell phenotype in a myocardin-dependent manner. This is functionally relevant in response to vascular injury (e.g., balloon injury-induced vessel lesion formation) in which YAP expression is induced [122]. Under these conditions YAP acts as a negative regulator of SRF-mediated gene transcription. However in another study YAP and MRTF-A were found to cooperate to promote GPCR/RhoA stimulated gene transcription and cellular proliferation [123] (Fig. 3c). Knockdown of YAP or MRTF-A blocked induction of *CCN1* (*Cyr61*) expression stimulated by S1P-mediated activation of GPCRs in glioblastoma cells. Like myocardin, MRTF-A was found to associate with YAP in co-immunoprecipitation experiments following GPCR stimulation. Functionally, both YAP and MRTF-A bind to the CCN1 promoter to drive S1P-stimulated glioblastoma cell proliferation [123]. Consistent with this, a recent paper by Cui et al. [93] reported that knockdown of either MRTF-A or YAP blocked cyclic stretch-stimulated spreading and proliferation of primary mouse embryonic fibroblasts on soft surfaces. Interestingly, knockdown of either YAP or MRTF-A impeded nuclear localisation of the other protein in response to cyclic stretching, though the mechanism of this regulation is yet to be elucidated.

More recently, two reports identified a link between MRTF and TAZ [124, 125]. MRTF/SRF signaling promotes *TAZ* gene expression and protein abundance downstream of activation by heregulin β1 in breast cancer cells [124]. Comparably, MRTF knockdown in a porcine kidney cell line resulted in significant down-regulation of TAZ mRNA and protein [125]. Similar to previous reports that found MRTFs could interact directly with YAP, Speight et al. [125] demonstrated that TAZ and MRTF associate, at least in part, by WW domain/PPxY-mediated interaction [126, 127]. Importantly however, the authors elegantly showed that despite their interaction, MRTF and TAZ translocate independently to the nucleus upon actin polymerization [125]. In fact, in an interestingly complex scheme of protein crosstalk, TAZ and MRTF reciprocally mitigate each other’s nuclear localisation and accumulation induced by low calcium (Fig. 3c). This observation is hypothesised to be mediated by TAZ-MRTF interaction, which may sequester both proteins in the cytoplasm. Furthermore, MRTF was found to up-regulate 14-3-3 expression, which is expected to increase cytoplasmic sequestration of both TAZ and YAP [125]. The crosstalk between these transcriptional co-factors is significant in light of the knowledge that interaction of TAZ and MRTF can have different transcriptional outcomes. Specifically, TAZ and MRTF antagonize each other on the α-SMA promoter, whilst synergizing on TEAD elements that are not located neat to a SRE/CArG sequence [125].

Heregulin β1 (a splicing isoform of neuregulin 1) is a soluble protein that binds to and activates the receptor protein tyrosine kinase ERBB4. Upon activation, the intracellular cytoplasmic domain (ICD) of ERBB4 translocates to the nucleus where it can activate transcription. Via a WW domain/PPxY-mediated interaction, YAP interacts with ERBB4 ICD to stimulate transcription [128]. This interaction, producing a YAP-TEAD-ERBB4 tripartite complex, was later shown to induce YAP target genes such as *CTGF*, and promoted YAP-dependent cell migration in response to neuregulin treatment in mammary carcinoma cells [129]. Interestingly, protein tyrosine kinases (including ERBB4) are principally involved in the formation of focal adhesions and rigidity sensing (reviewed in [130]). Knockdown of ERBB4 in cultured human fibroblasts significantly reduced rigidity-dependent cell polarization, characterised by reduced cell elongation and focal adhesion alignment, but with increased focal adhesion number, on both soft and rigid substrates [131]. These findings reveal that activation of ERBB4 via chemical (heregulin β1/neuregulin signaling) or mechanical (rigidity) cues can alter YAP/TAZ signaling via two different mechanisms. Hence ERBB4 should be considered to be a key regulator of YAP/TAZ activity.

As discussed above, MRTF associates with Smad3 to drive *slug* expression [27]. Intriguingly, Smad3 inhibits MRTF-dependent activation of the α-SMA promoter by reducing MRTF association with SRF [132] (Fig. 3c). TAZ has also been reported to cooperate with Smad3 to drive expression of *α-SMA*, and in an additional layer of complexity, treatment with TGFβ altered the relative interaction between MRTF, Smad3 and TAZ [125]. This is meaningful since TGFβ is a potent biochemical inducer of fibrogenesis, mediated by downstream MRTF signaling, thus the relative abundance of these multiple signaling mediators, in addition to the mechano- and chemical- stimuli detected by cells will precisely dictate the response at the level of gene transcription.

As another example of crosstalk between mechanosensing pathways, β-catenin was identified to be a positive regulator of MRTF signaling by alleviation of Smad3 inhibition via two mechanisms [133] (Fig. 3c). First, β-catenin competes with Smad3 for MRTF binding, freeing MRTF to associate with SRF. Second, β-catenin supresses Smad3-mediated recruitment of glycogen synthase kinase-3β to MRTF that leads to its ubiquitination and degradation, thus increasing MRTF protein stability [133]. Interestingly, YAP and β-catenin cooperate to regulate mechanical strain induced cell proliferation [134]. Cell cycle re-entry and subsequent progression from G1 to S phase are mediated by YAP- and β-catenin- signaling respectively, however inhibition of either is sufficient to block cellular proliferation as determined by Edu incorporation. Notably, treatment with inhibitors to block YAP activity (e.g., YAP1-TEAD inhibitory peptide or verteporfin) also blocked cell cycle entry evidenced by a marked reduction in Ki67 positive staining [134]. Thus, through different but complementary roles, YAP and β-catenin coordinate to regulate biological function (Fig. 3c).

## Other points of interest

In this review we have touched on some of the reports of crosstalk and cooperation of various mechanosensitive transcriptional activators either via physical association or regulation of gene expression. Important to note however is that DNA transcription is not an absolute requirement for a cell’s response to mechanical stimuli. Indeed experiments have shown that cell fragments devoid of a nucleus are mobile, able to migrate over surfaces and through basement membrane and endothelium towards a chemoattractant source [135, 136]. Furthermore, there is evidence to suggest that shedding of anucleate cytoplasmic fragments (microplasts) correlates with tumour cell invasiveness [137], suggesting that cell fragments may play a significant biological role, and could potentially be harnessed as vectors to deliver therapeutic agents. Localised force sensing and feedback mechanisms exist that enable cells, and even tiny cell fragments, to sense and respond to mechanical cues. Whilst the longer-term downstream effects of these events may still reach the cell nucleus to regulate gene expression, there are several examples of molecules and molecular complexes that can respond directly to mechanical stimuli, including adhesion complexes, the actomyosin network, and mechanosesitive ion channels (reviewed in [138]), which we will discuss briefly here.

Cells interact with each other and their environment via the formation of various adhesion complexes. Focal adhesions in particular have been shown to behave as individual mechanosensors. In response to applied force, focal adhesions exhibit directional assembly resulting in elongation [139], and this was found to be the result of stretching of several focal adhesion proteins exposing hidden binding sites for partner proteins. Similarly, strengthening of intercellular adherens junctions, mediated by protein clustering, is observed upon direct application of mechanical force [140]. Mechanical forces can also regulate the dynamics of the actomyosin network comprised of F-actin filaments cross-linked by the myosin II molecular motor. Load stabilises myosin in a state that sustains tension [141]. Furthermore, the elongation rate of formin mDia1 is increased up to two-fold by mechanical pulling, hypothesised to be due to the fact that pulling force favours the ‘open’ conformation, allowing an additional actin subunit to be added at the filament end [142]. Finally, mechanosensitive ion channels, also known as stretch-gated ion channels, respond directly to changes in cellular membrane tension by undergoing a conformational change to translate external physical stimuli into electrical signals. Other mechanosensitive channels are coupled to the cell cytoskeleton, thus movement of the cell relative to the ECM can also activate these channels. Flux of particular ions, such as Ca^2+^, induces a variety of cell responses including regulation of actin dynamics affecting cell contractility, mobility and adhesion formation (reviewed in [143]).

Crosstalk between the various mechanosensitive transcriptional activators discussed in this review can be seemingly straightforward: as in the direct binding of YAP/TAZ with myocardin/MRTF, or involve multiple competing and complimentary interactions between several factors: such as all combinations of SMAD or β-catenin with YAP, TAZ and MRTF. Whilst these are important and interesting examples of signaling crosstalk, they are not necessarily limited to direct protein-protein interactions. In the elegant study by Zanconato et al. [144] for example, the authors show that the YAP/TAZ/TEAD complex synergizes with the “classic proto-oncogene” AP-1 factors that are bound to composite *cis*-regulatory elements. Though AP-1 factors do not mediate YAP/TAZ DNA binding, nor was there evidence to suggest the main AP-1 proteins interact directly with YAP/TAZ, AP-1 factors jointly regulate a slew of YAP/TAZ/TEAD target genes that enhance YAP-dependent oncogenic activity [144]. Care should therefore be taken when examining mechanosensitive signaling pathways, remembering that they do not operate in isolation. Altering the expression or activity of even a single mediator will have far-reaching implications, and we predict the complexity will only increase, as these important new layers of signaling pathway integration are uncovered.

Further to that note, from a large scale analysis of somatic point mutations across 21 human cancer types myocardin was identified as a new oncogene that is mutated in cancer [145]. Interestingly, a cluster of nine mutations was identified within the region of myocardin that encodes the conserved PPxY motif that is responsible for YAP/TAZ/MYOCD interaction. It would be of interest to explore this finding to determine whether YAP/TAZ interaction with myocardin is compromised in these tumours, and whether this plays a role in their oncogenic phenotype, since if YAP and myocardin can no longer interact, their interaction with other mediators would be favoured to mediate different signaling outcomes. This study highlights the potential for studies of large sample size to detect previously undetected, yet highly relevant, cancer causing mutations that will help to guide our understanding of the complex interactions between known signal transduction pathways.

A feature of YAP signaling that is relatively unexplored, yet may yield significant insight into mechanotransduction mechanisms, is the potential differences between YAP splicing isoforms. There are at least eight reported isoforms of human YAP that are detectable as RNA in a range of human tissues [146]. Studies comparing various YAP isoforms have identified differences with regards to protein-protein interactions, e.g., with ERBB4 [128], AMOT [147], and p73 [59], as well as their relative transcriptional coactivator activities [128, 148]. Thus whether differential expression of YAP isoforms can influence mechanotransduction induced by mechanical cues, and whether this is linked to YAP-isoform specific interactions with other mechanosensitive mediators, remains to be determined.

## Conclusions

Cells within a tissue exist in a complex environment that is constantly changing. Cells need to be able to sense and respond accordingly to the multitude of signals they encounter, which includes mechanical cues such as pushing, pulling and shear stress. Regulation of gene transcription by actin dynamics is absolutely crucial to coordinate complex processes such as migration, mitosis, and intracellular trafficking. Transcription factors that form complexes with actin binding proteins, or bind directly to actin itself are going to be particularly responsive to actin dynamics. The MRTFs and Hippo pathway effectors YAP and TAZ are well-characterised examples of mechano-responsive transcription factors. As we learn more about the players and processes of actin dynamics we anticipate that new mechanotransducers will be identified. These discoveries will have important implications for understanding development and disease, and how these factors might be targeted therapeutically.

## Abbreviations

Ala: Alanine
AMOT: Angiomotin
Arg: Arginine
Asn: Asparagine
Asp: Aspartic acid
CCl_4_: Carbon tetrachloride
Cys: Cysteine
E: Embryonic day
ECM: Extracellular matrix
EMT: Epithelial–mesenchymal transition
GEFs: Guanine nucleotide exchange factors
Glu: Glutamic acid
GPCR: G protein-coupled receptors
H3K4: Histone H3 Lysine 4
hPSCs: Human pluripotent stem cell
ICD: Intracellular cytoplasmic domain
ISX: Isoxazole
JNK: c-Jun N-terminal kinase
LIMK1: LIM kinase-1
LPA: Lysophosphatidic acid
MMP: Matrix metalloproteinase
MRTF: Myocardin-related transcription factor
MYOCD: Myocardin
ROCK: Rho associated kinase
RTK: Receptor tyrosine kinase
S1P: Sphingosine 1-phosphophate
Ser: Serine
SRE: Serum response element
SRF: Serum response factor
STARS: Striated muscle activator of Rho signaling
TAZ: Transcriptional coactivator with PDZ-binding motif
TGFβ: Transforming growth factor-β
TNC: Tenascin-C
Val: Valine
YAP: Yes-associated protein
Yki: Yorkie
α-SMA: α-smooth muscle actin
